# Role of extracellular volume fraction determined by dual-layer spectral detector CT in predicting the tumor grade of pancreatic neuroendocrine neoplasms

**DOI:** 10.1007/s11604-026-01976-w

**Published:** 2026-05-12

**Authors:** Nobuhiro Fujita, Kosuke Tabata, Keisuke Ishimatsu, Yasuhiro Ushijima, Daisuke Okamoto, Noriaki Wada, Satoshi Makise, Nao Fujimori, Kohei Nakata, Masahiro Itoyama, Shinichi Aishima, Yoshinao Oda, Kousei Ishigami

**Affiliations:** 1 Department of Clinical Radiology, Graduate School of Medical Sciences, Kyushu University, 3-1-1 Maidashi, Higashi-ku, Fukuoka, 812-8582 Japan; 2 Department of Molecular Imaging and Diagnosis, Graduate School of Medical Sciences, Kyushu University, 3-1-1 Maidashi, Higashi-ku, Fukuoka, 812-8582 Japan; 3 Department of Medicine and Bioregulatory Science, Graduate School of Medical Sciences, Kyushu University, 3-1-1 Maidashi, Higashi-ku, Fukuoka, 812-8582 Japan; 4 Department of Surgery and Oncology, Graduate School of Medical Sciences, Kyushu University, 3-1-1 Maidashi, Higashi-ku, Fukuoka, 812-8582 Japan; 5 Department of Anatomic Pathology, Graduate School of Medical Sciences, Kyushu University, 3-1-1 Maidashi, Higashi-ku, Fukuoka, 812-8582 Japan; 6 Department of Scientific Pathology, Graduate School of Medical Sciences, Kyushu University, 3-1-1 Maidashi, Higashi-ku, Fukuoka, 812-8582 Japan

**Keywords:** Pancreatic neuroendocrine neoplasm, Dual-energy CT, Dual-layer spectral detector CT extracellular volume, Tumor grade

## Abstract

**Purpose:**

To evaluate the CT imaging features including extracellular volume (ECV) fraction measured by dual-layer spectral detector CT for differentiating G1 from G2 tumors in pancreatic neuroendocrine neoplasms (pNENs), as compared with single-energy CT (SECT).

**Materials and methods:**

This retrospective study included 45 patients with histologically confirmed pNENs (G1, *n* = 28; G2, *n* = 17) who underwent dynamic contrast-enhanced CT using a dual-layer spectral detector system. Attenuation values were measured on unenhanced and the equilibrium-phase 120-kVp equivalent CT images for tumor and the aorta, and SECT-ECV fraction was calculated. Iodine densities of the tumor and aorta were measured in the equilibrium phase, and dual-energy CT (DECT)-ECV fraction of the tumor was calculated. Histological tumor cell density and Ki-67 index were also assessed to evaluate the correlations with ECV fractions. Statistical analysis was performed to identify independent predictors of tumor grade and evaluate diagnostic performance using receiver operating characteristic (ROC) analysis.

**Results:**

DECT-ECV fractions were significantly lower in G2 tumors than in G1 tumors (*p* = 0.0045). G2 tumors tended to be larger than G1 tumors (*p* = 0.0591), while no other CT features were significantly different between G1 and G2 tumors. The multivariable analysis showed that only DECT-ECV fraction was significantly associated with tumor grade (*p* = 0.0115). ROC analysis for the DECT-ECV fraction showed an area under the curve of 0.756. DECT-ECV fraction demonstrated significant inverse correlations with tumor cell density (*ρ* = − 0.434, *p* = 0.0065) and Ki-67 index (*ρ* = − 0.440, *p* = 0.0025).

**Conclusion:**

Among the CT imaging findings, only DECT-ECV fraction showed a significant difference between G1 and G2 tumors. DECT-ECV fraction appears to be a useful imaging biomarker for differentiating G1 from G2 tumors in pNENs.

## Introduction

Pancreatic neuroendocrine neoplasms (pNENs) comprise a heterogeneous group of tumors with diverse biological behavior [1]. The World Health Organization (WHO) classification categorized pNENs into three grades (G1, G2, and G3) and neuroendocrine carcinoma (NEC), based on Ki-67 and mitotic count [2]. G3 pNENs and NECs are a distinct prognostic entity to G1–2 tumors and are typically associated with poorer outcomes [3]. In terms of imaging appearances, G3 pNENs and NECs tend to exhibit atypical imaging findings for pNENs (including ill-defined margins, heterogeneous hypovascular enhancement patterns, and pancreatic duct dilation) more closely resembling those of pancreatic ductal adenocarcinomas (PDACs), increasing the difficulty of differentiating G3 tumors or NECs from PDACs [4–6]. While the distinction between high-grade (G3/NEC) and low-grade (G1/G2) tumors is clinically vital, the preoperative differentiation between G1 and G2 tumors is also of significant clinical importance because G2 tumors exhibit more aggressive biological behavior and a poorer prognosis than G1 tumors [7]. In localized, well‑differentiated pancreatic neuroendocrine tumors, surgical resection is the standard of care; however, active surveillance may be considered for small, incidentally detected, non‑functional G1 lesions without high‑risk features, whereas G2 tumors of comparable size are generally regarded as stronger candidates for upfront surgery because of their higher malignant potential and increased risk of progression [8]. Despite these differences, G1 and G2 tumors often show overlapping appearances on conventional CT, ranging from typical hypervascularity to various atypical findings [9, 10]. Therefore, a quantitative and objective imaging biomarker is needed to distinguish G2 from G1 tumors.

Compared to conventional single-energy CT (SECT), dual-energy CT (DECT) facilitates more precise tissue discrimination by exploiting differences in X-ray attenuation at both high and low energies [11, 12]. DECT permits measurement of the extracellular volume (ECV) fraction without subtraction by generating separate iodine-density images in the equilibrium phase of contrast-enhanced CT [12, 13]. ECV fraction measured in the equilibrium phase of DECT has been shown to correlate strongly with extracellular space [14]. Considering the fact that G2 tumors exhibit greater tumor proliferative activity than G1 tumors [2], resulting in higher cellular density and lower extracellular space, we hypothesized that the ECV fraction of pNENs could serve as a useful imaging biomarker for tumor grading. However, to the best of our knowledge, no previous studies have evaluated the DECT-derived ECV fraction for grading pNENs. Therefore, the purpose of this study was to evaluate the value of CT imaging features, including the DECT-derived ECV fraction, for grading pNENs, with a particular focus on differentiating G1 from G2 tumors.

## Materials and methods

### Patients

**Fig. 1:**
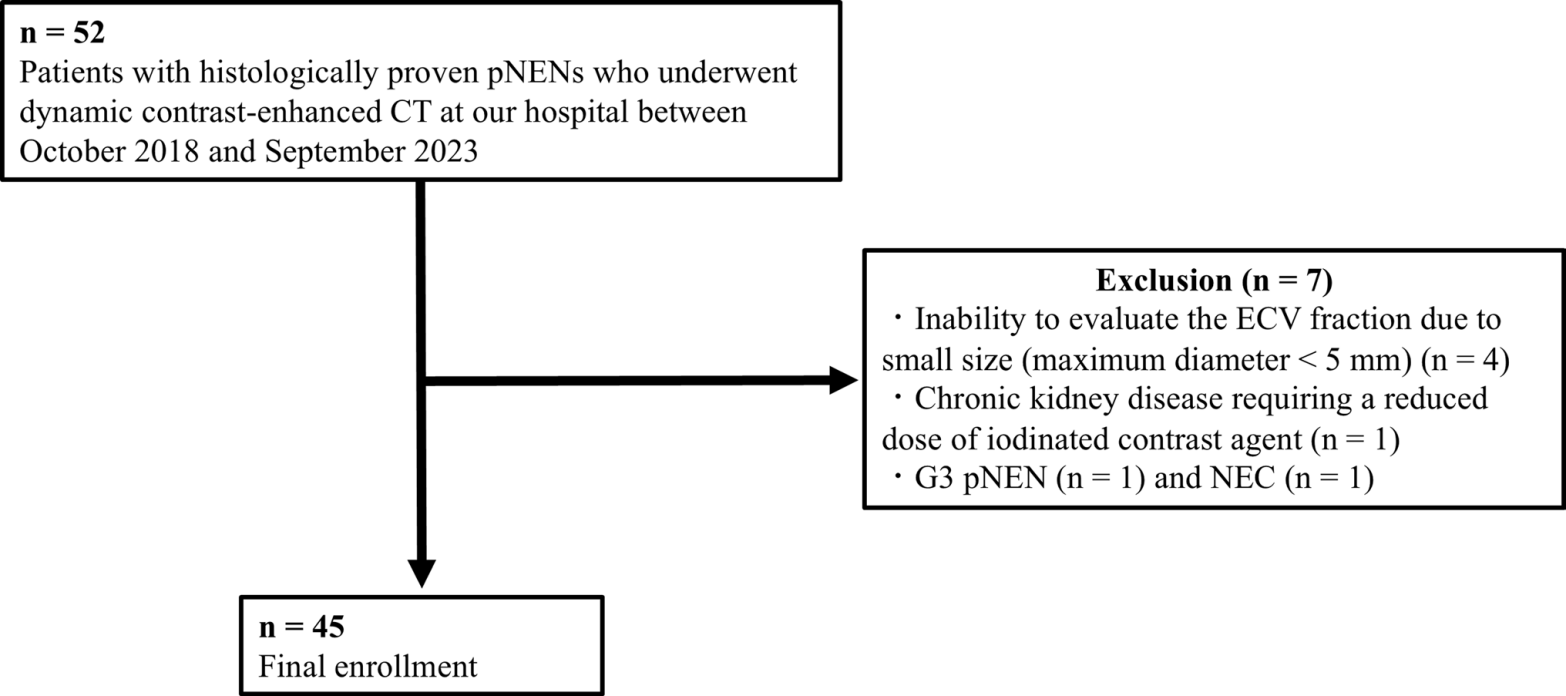
Flowchart of patient selection. NEN, neuroendocrine neoplasm; ECV, extracellular volume; NEC, neuroendocrine carcinoma

The study design was approved by our institutional review board, and the requirement for informed consent was waived due to its retrospective design. Eligible for inclusion in the study were 52 consecutive patients with histologically proven pNENs (surgical resection, *n* = 40; endoscopic ultrasonography-guided fine needle aspiration [EUS-FNA], *n* = 12) who underwent dynamic contrast-enhanced CT at our hospital between October 2018 and September 2023. None of the patients had received any prior treatments for pNENs before the CT examination. The exclusion criteria were as follows: 1) inability to evaluate the ECV fraction due to small size (maximum diameter < 5 mm), which resulted in tumor invisibility or an inability to accurately place an ROI (*n* = 4) [10], 2) chronic kidney disease requiring a reduced dose of iodinated contrast agent (*n* = 1), and 3) G3 pNEN (*n* = 1) and NEC (*n* = 1). Figure 1 shows a flow chart of patient selection. After applying these exclusions criteria, 45 patients remained and were retrospectively enrolled in the study.

Of these 45 patients with pNENs, 21 were women and 24 were men (median age, 56 years; interquartile range, 47.5–65.5 years). Pathological tumor grade was determined according to the WHO classification [2]. Twenty-eight tumors (62.2%) were classified as G1, and 17 tumors (37.8%) were classified as G2. In all patients, pathological tumor grade was determined using specimens obtained from the primary pancreatic tumor. The median interval between the CT examination and the histopathological diagnosis was 39 days (interquartile range, 18–61 days).

### CT protocols

Each patient underwent CT using a dual-layer spectral detector CT scanner with 64 detector rows (iQon Spectral CT; Philips Healthcare, Best, the Netherlands). Scanning was performed before and after administration of 600 mgI/kg of iodinated contrast material (Optiray 350 mg/mL, Guerbet, Villepinte, France), administered intravenously for 30 s. The four-phase dynamic study was performed as follows: the arterial phase was obtained using the bolus tracking technique, triggered during a single breath-hold after an increase of 100 HU in the descending aorta. Subsequently, the pancreatic parenchymal, portal venous, and equilibrium phases were acquired at fixed time points of 45, 60, and 240 s, respectively, after the initiation of contrast material injection. The imaging acquisition parameters were as follows: rotation time 500 ms, 128 × 0.625 mm collimator, tube voltage 120 kVp, automated tube current modulation, field of view 32 cm, image reconstruction thickness 1 mm, and spiral pitch 0.586. Conventional 120-kVp equivalent images were reconstructed using a standard B kernel with a hybrid iterative reconstruction technique (iDose^4^, level 4; Philips Healthcare). Spectral-based images were reconstructed at spectral level 0.

### Image analysis

Image analysis was performed independently by two radiologists (N.F. and K.T., with 21 and 10 years of experience, respectively, in abdominal imaging) who were blinded to the pathologic findings and to each other’s results. In the case of disagreement between the readers, a third radiologist (K.I., with 16 years of experience in abdominal radiology) analyzed the case, and the majority interpretation in qualitative binary assessment was used for categorical values. For continuous variables, the average of the two readers’ values was used. CT images were analyzed for the following features for each tumor: tumor maximum diameter, tumor margin (clear or irregular), upstream main pancreatic duct dilatation (> 3 mm), presence/absence of cystic or necrotic changes, and presence/absence of calcification [9, 10]. The enhancement pattern of the tumor on the pancreatic parenchymal, portal venous, and equilibrium phases was visually evaluated as hyper-attenuation or iso-/ hypo-attenuation relative to the pancreatic parenchyma [9]. The presence/absence of heterogeneous enhancement on parenchymal phase and of gradual enhancement on equilibrium phase were also evaluated [9, 10].

Attenuation values were measured on unenhanced and the equilibrium-phase 120-kVp equivalent CT images for pNEN and the aorta at the level of the pNEN by placing the regions of interest (ROIs) as large as possible. Care was taken to avoid necrosis or artifacts within the ROIs. An attempt was made to place the ROIs in identical sites for unenhanced and equilibrium phase CTs for each patient. ΔHU-tumor and ΔHU-aorta are HU in equilibrium phase minus HU in unenhanced CT of the tumor and the aorta, respectively. ΔHU-tumor/ΔHU-aorta was calculated, and SECT-ECV fraction (%) was calculated using the following formula: SECT-ECV fraction (%) = (1-hematocrit) × (ΔHU-tumor/ΔHU-aorta) × 100.

For the DECT-ECV fraction measurement, spectral-based image data from the equilibrium phase were postprocessed with a dedicated workstation (IntelliSpace Portal; Philips Electronics Japan) to generate iodine density images, as well as combined iodine density and conventional CT images. Iodine density was measured by placing a region of interest (ROI) over the tumor in a combined image. To ensure consistency, the ROIs for iodine density images were placed at the same locations as the ROIs used for the conventional 120-kVp equivalent images in the equilibrium phase. The iodine density in the aorta (I-aorta) was measured at the same level. Values of I-ratio and the DECT-ECV fraction were calculated using the following formula: I-ratio = I-tumor/I-aorta, DECT-ECV fraction (%) = (1-hematocrit) × I-ratio × 100.

For the tumor, the ROI size was 63.6 ± 81.6 mm^2^ (mean ± standard deviation (SD)) for Reader 1 (N.F) and 73.8 ± 118.8 mm^2^ (mean ± SD) for Reader 2 (K.T.). For the aorta, the ROI size was 113.3 ± 64.1 mm^2^ (mean ± SD) for Reader 1 (N.F) and 117.0 ± 73.0 mm^2^ (mean ± SD) for Reader 2 (K.T.). Hematocrit levels used for ECV fraction calculation were obtained from blood tests performed within a median of 6 days (interquartile range, 0.5–13.5 days) of the CT examination.

### Histological evaluation of tumor cell density

Tumor cell density was semi-quantitatively evaluated by two pathologists (M.I. and S.A., with 3 and 28 years of experience, respectively) who were blinded to all clinicopathological and imaging findings. Hematoxylin and eosin (H&E)-stained slides were available and reviewed for all 38 patients who underwent surgical resection in the study population. To ensure an accurate assessment of cellular density, care was taken to avoid areas of necrosis. The estimated ratio of tumor cells versus extracellular space was separated into quantiles (1–50%, 51–75%, and 76–100%) by scanning under low-power views (× 40). For those specimens with a relatively higher number of tumor, tumor cell density was assigned as 76–100% for specimens consisting predominantly of tumor cells, and 51–75% for the remaining specimens. For those specimens with a relatively higher area of extracellular space, a tumor cell density of 1–50% was assigned [15].

### Statistical analysis

Spearman’s rank correlation analysis was performed between SECT- and DECT-ECV fraction. Correlation between ΔHU-tumor/ΔHU-aorta and I-ratio, and SECT-ECV and DECT-ECV fractions, was analyzed using Bland–Altman analysis.

We used Fisher’s exact test for categorical variables and the Mann–Whitney *U* test for continuous variables to analyze differences in clinicopathological and CT imaging features between G1 and G2 tumors. Variables with *p* < 0.1 in the univariate analysis were entered into a stepwise multivariable logistic regression analysis to determine their independent relationship with tumor grade. Spearman’s rank correlation analysis was also performed between the histologically assessed tumor cell density and SECT- or DECT-ECV fraction, and Ki-67 index and SECT- or DECT-ECV fraction.

A receiver-operating characteristic (ROC) curve analysis was performed to determine the accuracy of imaging parameters for predicting G2. We calculated the area under the curve (AUC) to assess diagnostic performance. Standard definitions were used for calculation of sensitivity, specificity, accuracy, positive predictive value (PPV), and negative predictive value (NPV).

For categorical imaging findings, interobserver agreement between the two radiologists was evaluated using weighted κ statistics. For continuous CT imaging findings, intraclass correlation coefficients (ICCs) were calculated to evaluate inter-reader agreement. Correlation was considered excellent if the absolute value of κ-value or ICC was > 0.80, substantial if ≤ 0.80 to > 0.6, moderate if ≤ 0.6 to > 0.4, fair if ≤ 0.4 to > 0.2, and poor if ≤ 0.20. All statistical analyses were performed using JMP Pro version 15.1.0 (SAS Institute, Cary, NC) and MedCalc® Statistical Software version 20.210 (MedCalc Software Ltd, Ostend, Belgium; https:// www. medcalc. org; 2022). A p-value < 0.05 was considered significant.

## Results

The correlations between the clinicopathological findings between G1 and G2 tumors are summarized in Table 1. The Ki-67 index was significantly higher in G2 tumors (median, 4.8%; interquartile range, 4.0–6.5%) than in G1 tumors (median, 1.3%; interquartile range, 0.6–1.9%; *p* < 0.001). While tumor cell density showed a trend toward being higher in G2 tumors, the difference did not reach statistical significance (*p* = 0.0728).

**Table 1:** Comparison of clinicopathological findings between G1 and G2 pathological grades of pancreatic neuroendocrine neoplasm

	G1 (*n* = 28)	G2 (*n* = 17)	*P* value
Sex (male/female)	16/12	8/9	0.5512
Age*	55.5 (46.3–65)	57 (50.0–72.5)	0.3252
Ki-67 index (%)*	1.3 (0.6–1.9)	4.8 (4.0–6.5)	< 0.001
Tumor cell density (*n* = 38)			0.0728
1–50%	8 (34.8%)	1 (6.7%)	
51–75%	6 (26.1%)	3 (20.0%)	
76–100%	9 (39.1%)	11 (73.3%)	

^*^Values were expressed as median levels (interquartile range)

**Fig. 2:**
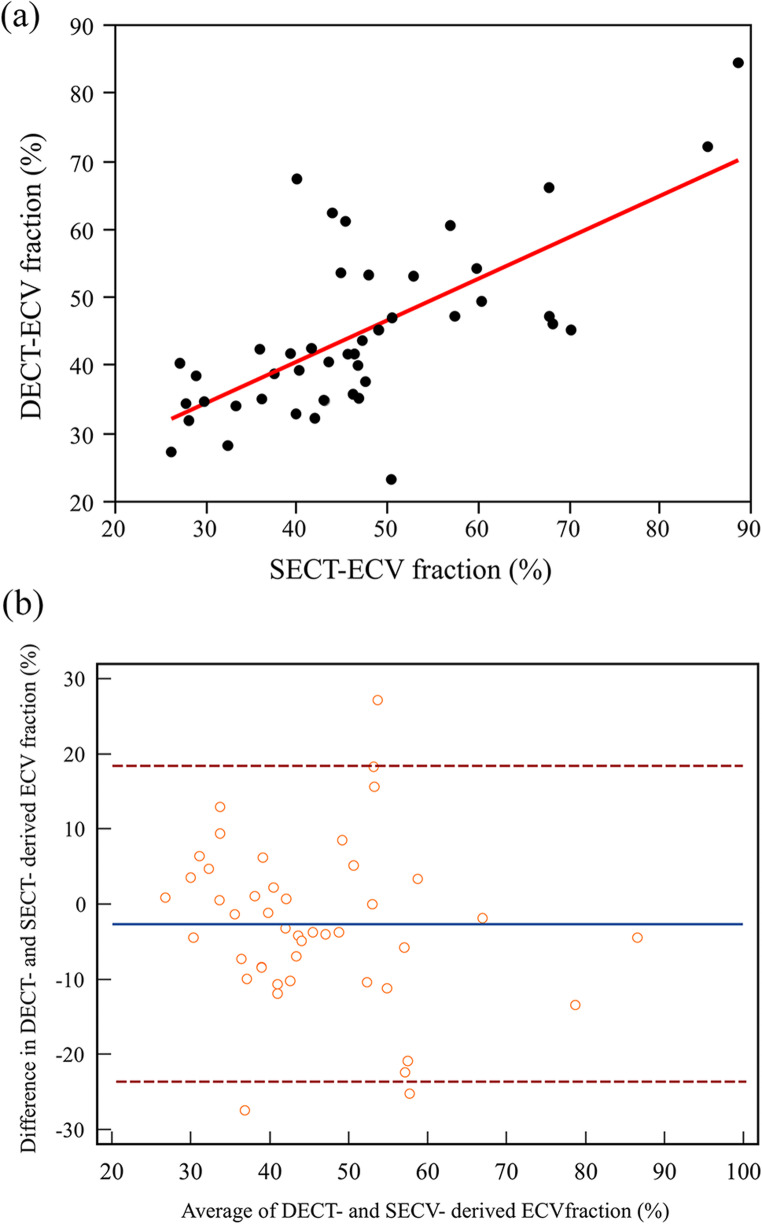
**a** Spearman’s rank correlation analysis showed a significant, moderate correlation between the SECT-ECV and DECT-ECV fractions (*ρ* = 0.649; 95% CI 0.439–0.792, *p* < 0.001). **b** Bland–Altman analysis of SECT-ECV and DECT-ECV fractions showed a small bias was obtained (− 2.67%) toward lower DECT-ECV fraction with 95% limits of agreement of − 23.75% and 18.40%

Spearman’s rank correlation analysis showed a significant, moderate correlation between the SECT-ECV and DECT-ECV fraction (*ρ* = 0.649; 95% CI 0.439–0.792, *p* < 0.001) (Fig. 2a). Bland–Altman analysis of SECT-ECV and DECT-ECV fractions showed a small bias was obtained (− 2.67%) toward lower DECT-ECV fraction with 95% limits of agreement of − 23.75% and 18.40% (Fig. 2b).

Table 2 summarizes comparisons of the CT imaging findings between G1 and G2 tumors. Tumor DECT-ECV fractions were significantly lower in G2 tumors (median, 35.7%; interquartile range, 34.1–41.6%) than in G1 tumors (median, 45.1%; interquartile range, 38.6–58.8%; *p* = 0.0045). G2 tumors (median, 20 mm; interquartile range, 11.5–50 mm) tended to be larger than G1 tumors (median, 14 mm; interquartile range, 9–22 mm; *p* = 0.0591). No significant difference was observed in the SECT-ECV fractions between G1 and G2 tumors (*p* = 0.4680).

**Table 2:** Comparison of CT imaging findings between G1 and G2 pathological grades of pancreatic neuroendocrine neoplasm

CT imaging finding	G1 (*n* = 28)	G2 (*n* = 17)	*P* value
Tumor diameter (mm)*	14 (9–22)	20 (11.5–50)	0.0591
Tumor margin (clear/irregular)			0.1438
Clear	27 (96.4%)	14 (82.4%)	
Irregular	1 (3.6%)	3 (17.6%)	
Main pancreatic duct dilatation			0.1438
(+)	1 (3.6%)	3 (17.6%)	
(−)	27 (96.4%)	14 (82.4%)	
Cystic or necrotic change			
(+)	3 (10.7%)	2 (11.8%)	1.0000
(−)	25 (89.3%)	15 (88.2%)	
Calcification			
(+)	3 (10.7%)	2 (11.8%)	1.0000
(−)	25 (89.3%)	15 (88.2%)	
Enhancement pattern			
Pancreatic parenchymal phase			0.7109
Hyper-attenuation	23 (82.1%)	13 (76.5%)	
Iso/hypo-attenuation	5 (17.9%)	4 (23.5%)	
Portal phase			
Hyper-attenuation	21 (75.0%)	13 (76.5%)	1.0000
Iso/hypo-attenuation	7 (25.0%)	4 (23.5%)	
Equilibrium phase			
Hyper-attenuation	16 (57.1%)	10 (58.8%)	1.0000
Iso/hypo-attenuation	12 (42.9%)	7 (41.2%)	
Heterogeneous enhancement			0.7439
(+)	8 (28.6%)	6 (35.3%)	
(−)	20 (71.4%)	11 (64.7%)	
Gradual enhancement pattern			1.0000
(+)	1 (3.6%)	1 (5.9%)	
(−)	27 (96.4%)	16 (94.1%)	

SECT-ECV fraction (%)*	45.2 (40.0–57.4)	45.7 (34.8–48.7)	0.4680
DECT-ECV fraction (%)*	45.1 (38.6–58.8)	35.7 (34.1–41.6)	0.0045

^*^Values were expressed as median levels (interquartile range). SECT-ECV, single energy CT-derived extracellular volume; DECT-ECV, dual energy CT-derived extracellular volume

The multivariable model including tumor diameter and DECT-ECV fraction showed that DECT-ECV fraction was significantly associated with tumor grade (odds ratio, 1.1294; 95% confidence interval (CI), 1.0278–1.2412; *p* = 0.0115) (Table 3).

**Table 3:** CT imaging findings associated with pathological grade of pancreatic neuroendocrine neoplasms: multivariable analysis

CT imaging finding	*P* value	Odds ratio	95% CI
Tumor diameter (mm)	0.0651	0.9475	0.8948–1.0034
DECT-ECV fraction (%)	0.0115	1.1294	1.0278–1.2412

DECT-ECV, dual energy CT-derived extracellular volume; CI, confidence interval

**Fig. 3:**
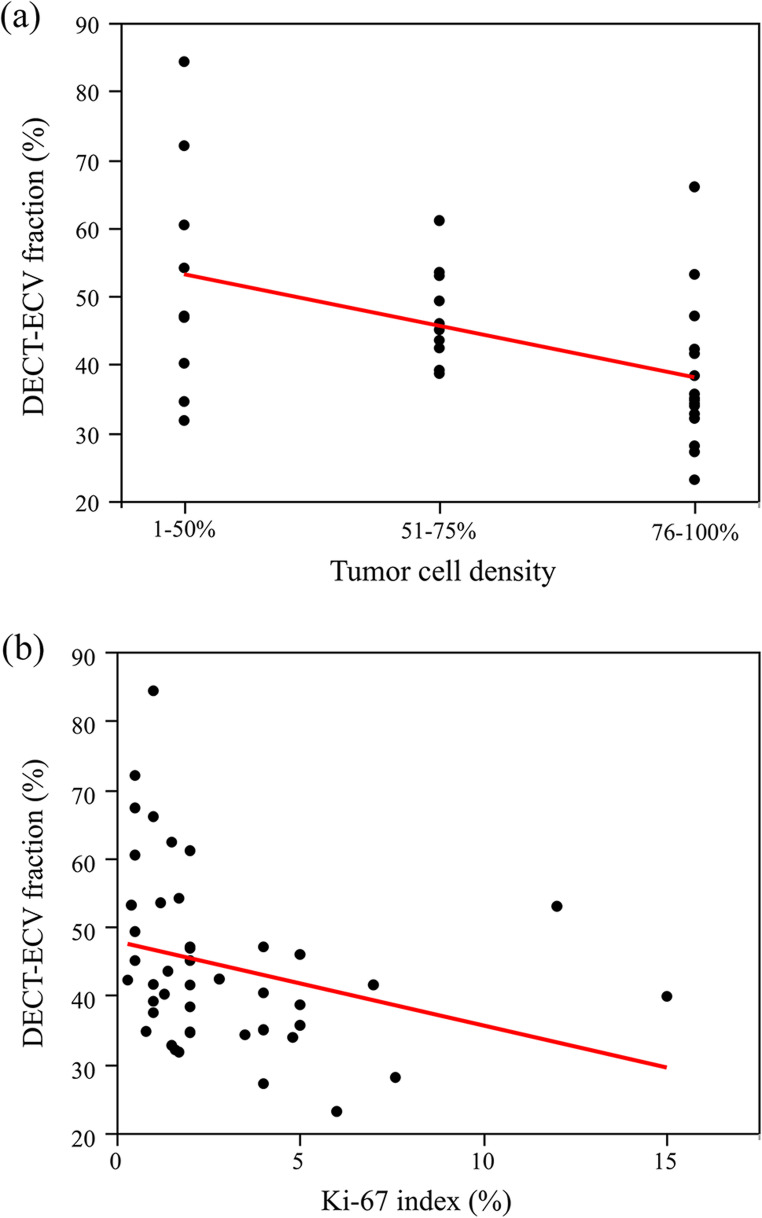
**a** Correlation between tumor cell density and DECT-ECV fraction. A significant, moderate inverse correlation between the DECT-ECV fraction and tumor cell density (*ρ* = − 0.434, *p* = 0.0065). **b** Correlation between the Ki-67 index and DECT-ECV fraction. A significant, moderate inverse correlation was observed between the Ki-67 index and DECT-ECV fraction (*ρ* = − 0.440, *p* = 0.0025)

Spearman’s rank correlation analysis showed a significant, moderate inverse correlation between the DECT-ECV fraction and both tumor cell density (*ρ* = − 0.434; 95% CI − 0.662 to − 0.132, *p* = 0.0065) (Fig. 3a) and Ki-67 index (*ρ* = − 0.440; 95% CI − 0.650 to − 0.169, *p* = 0.0025) (Fig. 3b). No significant correlations were observed between the SECT-ECV fraction and either tumor cell density (*ρ* = − 0.306; 95% CI − 0.570 to − 0.015, *p* = 0.0620) or Ki-67 index (*ρ* = − 0.154; 95% CI − 0.428 to 0.146, *p* = 0.3117).

**Fig. 4:**
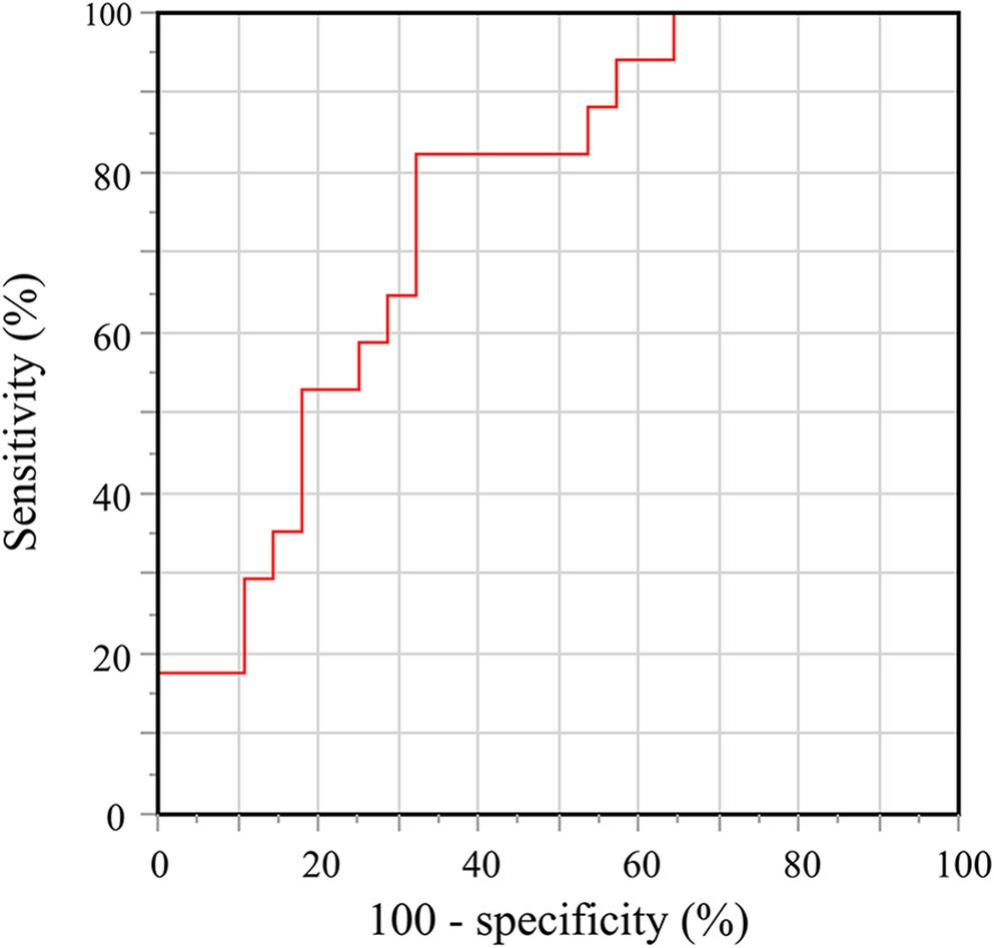
Receiver-operating characteristic analysis of DECT-ECV fraction for differentiating G1 and G2 tumors. The area under the curve was 0.756

DECT-ECV fraction demonstrated the diagnostic performance for tumor grade, with an AUC of 0.756 (95% CI 0.615–0.898) (Fig. 4). The optimal cutoff value for identification of G2 tumors was 41.6% for DECT-ECV fraction, and its sensitivity, specificity, accuracy, PPV and NPV were 82.4% (95% CI 56.6–96.2%), 67.9% (95% CI 47.6–84.1%), 73.3% (95% CI 58.1–85.4%), 60.9% (95% CI 46.5–73.6%), and 86.4% (95% CI 68.7–94.8%), respectively.

**Fig. 5:**
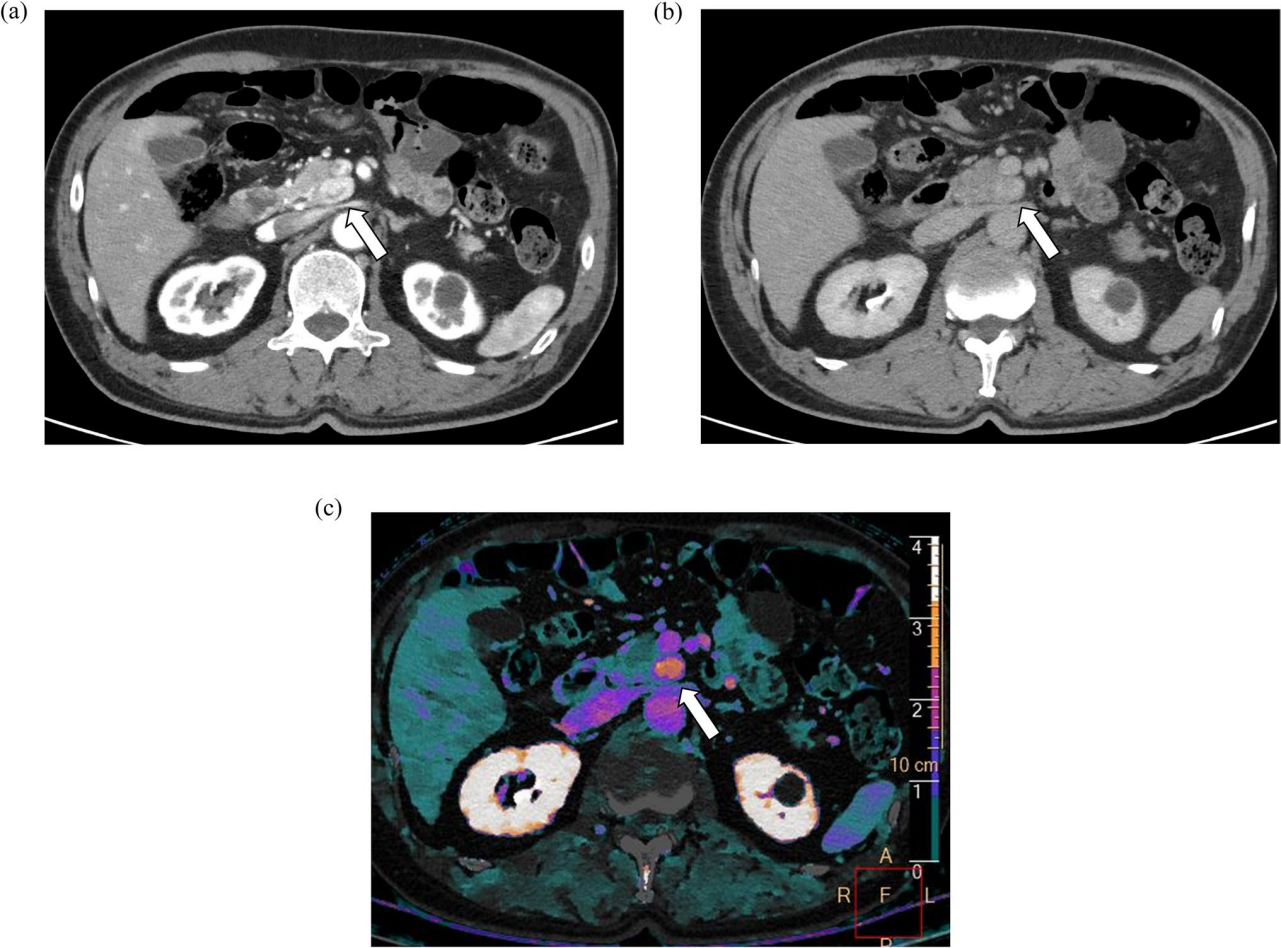
A 65-year-old man with a pancreatic neuroendocrine neoplasm (G1) in the pancreatic head (arrows). Dynamic CT imaging reveals a hyper-attenuating tumor relative to pancreatic parenchyma in the pancreatic parenchymal (**a**) and equilibrium phases (**b**). Fusion image of the iodine density and equilibrium phase images (**c**). The DECT-ECV fraction was 65.9%. The Ki-67 index was 1.0%, and histological tumor cell density was classified as 51–75%

**Fig. 6:**
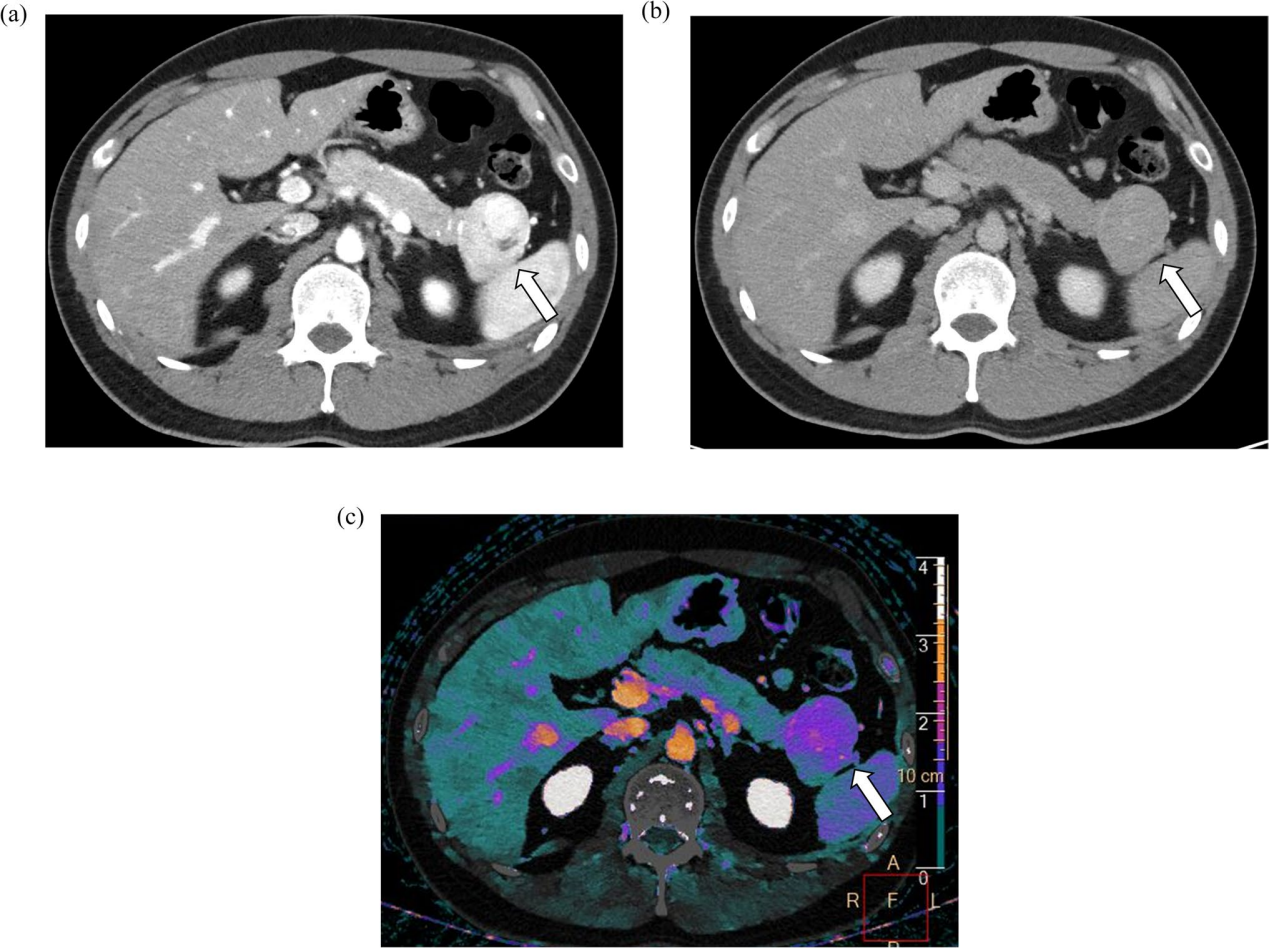
A 32-year-old man with a pancreatic neuroendocrine neoplasm (G2) in the pancreatic tail (arrows). Dynamic CT imaging reveals a hyper-attenuating tumor relative to pancreatic parenchyma in the pancreatic parenchymal phase (**a**). In the equilibrium phase, the tumor shows iso-attenuation relative to pancreatic parenchyma (**b**). Fusion image of the iodine density and equilibrium phase images (**c**). The DECT-ECV fraction was 33.9%. The Ki-67 index was 4.8%, and histological tumor cell density was classified as 76–100%

Representative patient images are shown in Figs. 5 and 6.

There was excellent interobserver agreement for tumor diameter (ICC = 0.9887), enhancement pattern in the pancreatic parenchymal (κ = 0.9275) and equilibrium phases (κ = 0.8643), and for DECT-ECV fraction (ICC = 0.8556). For other CT imaging findings, interobserver agreement was substantial (κ = 0.6009–0.7887) (Table 4).

**Table 4:** Intraclass correlation coefficient and κ values of imaging findings

		95% CI
Tumor diameter (mm)	0.9887*	0.9796–0.9938
Tumor margin (clear/irregular)	0.6342**	0.2619–1.0000
Main pancreatic duct dilatation	0.7256**	0.3639–1.0000
Cystic or necrotic change	0.6897**	0.3612–1.0000
Calcification	0.7273**	0.3718–1.0000
Enhancement pattern		
Pancreatic parenchymal phase	0.9275**	0.7875–1.0000
Portal phase	0.6600**	0.4116–0.9083
Equilibrium phase	0.8643**	0.7162–1.0000
Heterogeneous enhancement	0.6009**	0.3539–0.8479
Gradual enhancement pattern	0.7887**	0.3885–1.0000

SECT-ECV fraction (%)	0.7870*	0.5780–0.8925
DECT-ECV fraction (%)	0.8556*	0.7373–0.9207

^*^Intraclass correlation coefficients. **κ value. CI, confidence interval; SECT-ECV, single energy CT-derived extracellular volume; DECT-ECV, dual energy CT-derived extracellular volume

## Discussion

In the present study, the DECT-ECV fractions of G2 pNENs were significantly lower than those of G1 tumors. DECT-ECV fraction showed fair discrimination between G1 and G2 tumors (AUC = 0.756) [16, 17], with sensitivity and specificity of 82.4% and 67.9%, respectively. Consistent with the WHO grading system, the Ki-67 index was significantly higher in G2 tumors in our cohort. While the difference did not reach statistical significance, tumor cell density showed a trend toward being higher in G2 tumors. These results likely reflect the higher degree of tumor proliferation for G2 pNENs than for G1 pNENs, leading to higher cellular density and lower extracellular space, and consequently a lower ECV fraction. Our histological assessment supported this, as a significant inverse correlation was found between tumor cell density and the DECT-ECV fraction, confirming that the ECV fraction accurately reflects the microscopic density of tumor cells. Additionally, a significant inverse correlation was found between the Ki-67 index and DECT-ECV fraction, suggesting that the DECT-ECV fraction reflects the continuous spectrum of tumor proliferative activity. It has been reported that decreased apparent diffusion coefficient (ADC) values, which also reflect high cell density and decreased extracellular space, correlate with higher grades of pNENs [10, 18–21]. We did not evaluate MRI findings such as ADC values in this study, for the reasons that MRI was not performed in all cases and different MRI scanners were used among patients. However, the ECV fraction, like ADC values, likely reflects differences in cell density and extracellular space in pNENs, and may serve as a useful parameter for differentiating G1 from G2 tumors. Iwaya et al. reported that the ECV fraction measured by conventional SECT showed an inverse relationship with tumor grade in pNENs [22]. ECV fraction measurements using SECT remain limited by the requirement for precontrast CT (increasing radiation dose) and the possibility of misregistration between pre- and postcontrast images. Herein, we present the clinical significance of ECV fraction measured by DECT for predicting tumor grade in pNENs.

For the prediction of tumor grade in pNENs, tumor size has been shown to be strongly associated with grade, even among G1/G2 tumors [9, 19, 23]. In addition, previous studies have shown that tumor margin, the presence of a cystic component, and attenuation/signal intensity in dynamic CT/MRI correlated with tumor grade in pNENs [9, 10, 19, 23, 24]. In the present study, G2 tumors tended to be larger than G1 tumors, but the difference was not significant in multivariate analysis. Other CT imaging findings were not significantly different between G1 and G2 tumors. Interestingly, while visual assessment of the equilibrium phase enhancement pattern did not significantly differ between G1 and G2 tumors, the DECT-ECV fraction was a significant predictor. This discrepancy likely arises because the DECT-ECV fraction provides a normalized, quantitative measure of the extracellular space by accounting for aortic iodine concentration and hematocrit, whereas visual assessment is qualitative and influenced by systemic factors and the variability of the pancreatic parenchyma used as a reference. Further examination with a larger number of patients is needed; however, our results suggest that DECT-ECV fraction may be a more useful biomarker for predicting tumor grade in pNENs than other imaging findings, especially in G1 and G2 tumors.

An important consideration is that ECV fraction is highly correlated with the amount of desmoplastic stroma or fibrosis. While qualitative enhancement patterns often fail to capture subtle interstitial changes, the DECT-ECV fraction provides a quantitative measure that can more sensitively reflect the microscopic extracellular space. In support of this, our histological evaluation demonstrated a significant inverse correlation between the DECT-ECV fraction and tumor cell density. This indicates that the lower ECV fraction observed in G2 tumors primarily reflects increased cellular density and a corresponding reduction in the extracellular space. These observations could be specifically attributable to the fact that our study focused on G1 and G2 tumors, where the difference in cellularity between these two grades likely served as a primary determinant of the ECV fraction. However, when assessing ECV fraction using DECT, the potential influence of fibrosis cannot be entirely dismissed and should be considered in future studies, especially with larger cohorts. Notably, patients with G3 pNEN (*n* = 1) and NEC (*n* = 1) were excluded from this study, and our results are based only on G1 and G2 pNENs. Future studies with a larger patient cohort are needed to validate whether our results are applicable to higher-grade tumors. However, considering that G2 tumors also show more aggressive biological behavior and poorer prognosis compared with G1 tumors [7], our results may serve as a critical first step toward incorporating these quantitative tools into routine clinical practice for improved patient care.

SECT-ECV and DECT-ECV fraction showed a moderate correlation (*ρ* = 0.649, *p* < 0.001) and the mean bias was small (-2.67%). However, the wide limits of agreement (− 23.75% to 18.40%) suggest individual variation between SECT and DECT in measuring ECV. Notably, while the DECT- ECV fraction showed a significant difference between G1 and G2 tumors, the conventional SECT- ECV fraction failed to demonstrate a significant difference. Furthermore, the DECT-ECV fraction correlated significantly with both tumor cell density and the Ki-67 index, whereas the SECT-ECV fraction did not show such significant correlations. These results suggest that the DECT approach, which directly quantifies iodine density by isolating the iodine signal, provides a more accurate reflection of the extracellular space than the conventional Hounsfield unit-based SECT approach. SECT-ECV fraction measurements are often limited by the requirement for precontrast images and are susceptible to potential misregistration and beam-hardening artifacts, which may have masked the subtle differences between G1 and G2 tumors in our cohort.

DECT is an emerging and valuable tool for characterizing pancreatic diseases including pNENs [25]. It provides quantitative parameters such as iodine concentration (IC) and effective atomic number (Z_eff_), which can help differentiate between distinct pathological entities. Wang et al. showed that IC, Z_eff_, and portal venous phase CT attenuation were independent predictors for distinguishing pNEC from pNENs [26]. Li et al. reported that normalized λHU in the arterial phase and portal venous phase was an important variable for distinguishing non-low-grade pNENs (G2, G3) from low-grade pNENs (G1), and that DECT could be used to differentiate between the benign and malignant nature of pNENs [27]. However, to the best of our knowledge, no reports have evaluated the value of ECV fraction derived from DECT for grading pNENs. This study aimed to investigate the utility of ECV fraction derived from DECT for the preoperative grading of pNENs, and demonstrated its clinical significance for predicting tumor grade in pNENs. Future studies are needed to validate these findings in larger patient populations and to explore whether DECT-derived parameters can be integrated into clinical workflows to improve the prediction of tumor grade, and consequently treatment planning and prognosis prediction in pNENs.

There are several limitations to this study. First, this was a retrospective single-center investigation with a relatively small number of patients. However, according to power analysis for the Mann–Whitney *U* test of DECT-ECV fraction (*n* = 45), the statistical power was 0.9046 for an effect size of 1.0281, which is higher than the conventionally accepted standards for study power (0.8) [28]. Second, we performed semi-quantitative histological evaluation of tumor cell density. Histologically, tumor tissue volume is primarily the sum of the cellular component and the extracellular space; therefore, evaluating tumor cell density inversely reflects the extracellular space. However, more detailed digital image analysis of automated cell counting or direct quantification of fibrosis might provide a more precise correlation with the ECV fraction. Third, we did not conduct a comparison with MRI, because MRI was not performed in all cases. Fourth, a portion of our study population (12/45, 26.7%) was diagnosed via EUS-FNA rather than surgical resection. Because EUS-FNA samples may not always capture the Ki-67 'hot spot,' there is a possibility of underestimating the tumor grade compared to surgical specimens. This potential for misclassification might have affected the accuracy of our reference standard. Fifth, considering that pNENs might be a biologically heterogeneous group of tumors, mean ECV fractions might not fully capture intratumoral variability or focal high-grade ‘hot spots’ of Ki-67 index. To address this, we preliminarily evaluated the SD of attenuation values in the equilibrium phase and I-tumor of pNENs as an indicator of heterogeneity; however, no significant difference was observed between G1 and G2 tumors (data not shown). This result suggests that while heterogeneity exists, its impact on our differentiation between grades using mean values might be limited in this cohort. Nevertheless, future studies using methods, such as whole-tumor histogram analysis or radiomics-based texture analysis, are needed to further explore the diagnostic value of tumor heterogeneity. Sixth, the DECT-ECV fraction measurements in this study were obtained using a specific dual-layer spectral detector CT system. Since ECV fractions may vary depending on vendor-specific spectral acquisition and reconstruction algorithms, our proposed cutoff values require external validation across multiple DECT platforms to ensure generalizability. Finally, our results have limited diagnostic performance and clinical contribution due to the overlap in imaging characteristics between G1 and G2 tumors. However, we consider that this study will lead to future development of pNEN assessment using DECT.

In conclusion, among the CT imaging findings, only DECT-ECV fraction showed a significant difference between G1 and G2 tumors. DECT-ECV fraction appears to be a useful imaging biomarker for differentiating G1 from G2 tumors in pNENs.
